# Supplementary material for: Are carbon emissions trading and green financial instruments synergistic? -Comprehensive quantitative research based on content analysis
Source: PLoS One. 2024 Mar 7;19(3):e0298601. doi: 10.1371/journal.pone.0298601 (PMC10919601; doi:10.1371/journal.pone.0298601)
Supplement: S1 Data — (ZIP) [file pone.0298601.s001.zip › Data/地级市Z_F工作报告.zip!/╕ú╜¿/╚¬╓▌╩╨/└┤╫╘╣1⁄2╓┌║┼í╛┬φ┐╦╩2╛▌═°í┐_2004.docx]

各位代表：

现在，我代表泉州市人民政府向大会作工作报告，请予审议，并请政协委员和列席人员提出意见。

一、2003年政府工作回顾

2003年是全市人民在全面建设小康社会伟大征程上阔步前进的第一年。在省委、省政府和市委领导下，我市各级政府认真贯彻党的十六大、十六届三中全会精神，深入开展"泉州人的再思考"活动，奋力抗击非典疫情，努力克服严重干旱和缺电等困难，抢抓发展机遇，扎实推进经济、文化、城市、民生和政府建设，致力全面建设小康社会，经济社会持续快速协调健康发展。全年实现地区生产总值1380.11亿元，比增14%，经济总量继续保持全省第一；财政总收入突破百亿元大关，达105.01亿元，比增22.8%，其中，地方财政一般预算收入50.03亿元，比增26.5%。我市荣获国际花园城市、全国园林绿化先进城市、全国科技进步先进城市"四连冠"、全国双拥模范城"五连冠"等称号。市十三届人大四次会议确定的各项目标任务顺利完成。

推进项目带动和载体建设，综合经济实力进一步增强。五大基地建设扎实推进。关系全市经济社会发展大局的炼化一体化、泉南沿海大通道、南埔电厂等七大龙头项目建设启动。全社会固定资产投资288.15亿元，增长24.1%。三次产业结构进一步优化，比例由2002年的6.8：52.8：40.4调整为6.1：53.3：40.6。农业产业化稳步推进，"252"、"4121"、"1168"农业特色基地建设取得新成果。工业经济主导作用增强，全年实现增加值657.97亿元，增长17.3%，对经济增长的贡献率达61.3%。第三产业实现增加值560.72亿元，增长11.4%。消费需求进一步扩大，汽车、住房、通讯等消费热点突出，社会消费品零售总额（新口径）409.33亿元，增长12.1%。成功举办第五届泉州旅游节，组织评选"泉州十八景"，全市旅游总收入79.9亿元，增长9.2%。重视发展会展经济，举办福建省网上投洽会、泉州侨乡商品（乌鲁木齐）展洽会等一批大型商贸活动，签订合同交易金额103.18亿元。交通、电信、电力等进一步发展。泉州晋江机场客货吞吐量分别增长29.3%和23.9%，泉州港货物和集装箱吞吐量分别增长18.1%和50.2%。对外对台经贸合作取得新进展。全市出口商品总值（海关口径）19.16亿美元，比增24.9%，实际利用外资9.94亿美元，比增21.0%。新批台资企业59家，台商实际到资5269万美元。县域经济充满活力。晋江、石狮、惠安、南安继续入选"中国最发达100名县（市、区）"，上述四县（市）及安溪进入"全国国内生产总值位列前100名的县（市、区）"；鲤城、晋江、惠安、德化分别被评为"中国民间工艺品之都"、"中国纺织产业基地"、"中国石雕之都"、"中国瓷都"。非公有制经济快速发展。制订《进一步提升民营经济发展水平的若干意见》，非公有制企业体制技术管理创新加快。惠泉啤酒股票正式发行，凤竹纺织、七匹狼实业分别通过中国证监会审核和预审。新组建行业协会（商会）17家。新增中国名牌产品2项、国家免检产品26项，新通过ISO9000等国际标准管理体系认证企业443家。财税金融服务地方经济能力增强。至年底金融机构各项人民币存款余额1023.21亿元，贷款余额653.38亿元，分别增长24.8%和34.9%。

狠抓各项防控措施落实，抗击非典取得重大胜利。面对突如其来的非典疫情，全市各级在市委的坚强领导下，齐心协力，有为处置，在全省率先制订工作预案，突出抓重点人群防控、抓疫情报告网络和应急队伍建设，全市上下形成了群众参与、以防为主、全面覆盖的防控工作格局，实现了省委、省政府提出的"三无"目标。在应对非典的同时，相继出台一系列扶持企业的措施，努力把非典对经济发展的负面影响降到最低程度，保持了全市社会稳定和经济健康发展。

加快科教兴市步伐，社会各项事业全面发展。 "全国技术创新工程试点城市"等四大国家级科技示范工程扎实推进，268项国家、省、市级科技项目顺利实施，新增国家、省级高新技术企业23家。鲤城、洛江、泉港、晋江、石狮、南安、惠安、德化被评为全国科技进步先进县（市、区）。市高新技术孵化基地建设步伐加快，新增孵化企业20家。新建石狮、南安两个博士后工作站。出台《关于加快普及高中阶段教育和发展职业教育的决定》，高中阶段招生数增长13.8%，高考录取数增长12%。泉州高教发展中心奠基，泉州师院东海校区二期工程启动，泉州医科高专、福建电力职业技术学院、德化陶瓷学院获准招生，泉州经贸职业学院完成申报工作。民办教育快速发展，全市民办学校在校生增长21.4%。文化弘扬取得新成效。成功举办中国民系（闽南）文化节暨第二届中国泉州"海上丝绸之路"文化节、纪念施琅暨清廷统一台湾320周年等一系列活动。"海丝"申报"世界文化遗产"列入预备清单，保护规划获省政府批准。"南音"申报"人类口头及非物质遗产代表作"完成文本和专题片报送。梨园戏《董生与李氏》剧目入选国家舞台艺术精品工程，一批文艺作品获第12届伊朗国际传统戏剧节"金小丑奖"和全国首届"红梅奖"、第2届"金狮奖"、第3届"蒲公英奖"、第12届"群星奖"等奖项。梨园戏、南音赴法国参加中法文化年展演，泉州文化节在中东成功举办。市博物馆新馆、伊斯兰文化陈列馆等一批文化设施竣工。各项社会事业同步推进。卫生事业三项改革继续深化，公共卫生体系建设得到加强，创建国家卫生城市通过国家考核。成功举办第七届市运会，我市被评为全国残疾人体育工作先进单位。广电事业改革发展取得新成效。社会科学、文学艺术、新闻出版进一步创新和繁荣，民族宗教、外事侨务、妇女儿童、老龄、气象、防震减灾、档案、地方志等工作取得新进展。精神文明建设得到加强。"道德新风与你同行"活动深入开展，"诚信泉州"建设取得新成效。一批精神文明建设先进单位和个人受省委、省政府表彰。深入贯彻国防建设与经济建设协调发展方针，军事斗争准备扎实推进，国防动员、民兵、预备役和双拥共建等工作取得新成绩。

立足全省三大中心城市定位，城市规划建设管理迈出新步伐。提高城市规划水平，完成环泉州湾城市群概念规划方案和泉州市近期建设规划编制，完善洛秀、北峰、城东组团分区规划，开展清源山风景名胜区总体规划修编。确定市行政中心新址。加快城市建设步伐。中心城市构架进一步撑大，建成区面积达58.2平方公里，比上年扩大5平方公里。全市95个城建重点项目进展顺利。大坪山隧道、后渚大桥、江滨北路、城西环路、城北环路等一批项目竣工，中心城市进出通道首批拓改项目进展顺利，江南、洛秀、东海、池店等组团开发建设全面展开。古城保护力度加大，城南片区保护整治基本完成。泉州名城保护与建设获中国人居环境范例奖。加大城市管理力度。城市绿化、美化、亮化工作取得进展，市区主要道路、主要景观地带、主要建筑物首期"夜景工程"基本完成。中心城市建成区新增绿地185.3公顷，绿化覆盖率达32.3%。市区旧城拓改片区房产证办结率达99.3％。市区交通、市容市貌和环境卫生得到整治，市民住行条件继续得到改善。"镇改街道、村改社区"力度加大，鲤城、丰泽居民全部纳入城市管理。全市共招标拍卖、挂牌出让国有土地使用权109宗，国土资源有形市场建设初具规模。各地加强县城、中心城镇规划建设管理和改旧建新工作，全市城镇建设"四层联动"格局初步形成。全市城市化水平达45.9%，比上年提高4.6个百分点。

切实关注民生质量，人民生活水平继续提高。城镇居民人均可支配收入11155元，增长10.4%；农民人均纯收入5195元，增长6.5%。全市36%的县（市、区）、35.3%的乡镇、45.7%的村达到宽裕型小康标准。农村税费改革扎实推进，农电"两改"基本完成，全年减轻农民负担3.5亿元。市级落实帮扶项目160个、资金2670万元。实施"造福工程"，搬迁安置1000人。转移农村劳动力3.45万人。出台实施《泉州市保障外来务工人员合法权益暂行规定》。养老、失业、医疗、工伤、生育保险覆盖范围进一步扩大，城乡居民最低生活保障制度进一步落实。新增就业人数14.07万人，城镇登记失业率1.06%。创建生态市和国家环保模范城市稳步推进，晋江上游和山美水库水资源整治保护取得成效，晋江流域三类水质达标率97.4%，市区饮用水源水质达标率97%。人口与计划生育工作水平提高，全市人口出生政策符合率92.5%，计生合格和基本合格村率92.7%。推进社会治安综合治理，深化"严打"整治斗争，实现"一升一降"目标，综治检查考评名列全省首位。安全生产专项整治深入开展，安全生产形势总体平稳。整顿规范市场经济秩序成效明显。

强化执政为民观念，政府自身建设得到加强。"四五"普法深入开展，依法治市扎实推进。深化行政事业单位机构改革和人事制度改革，全市共分流机关干部职工1418人，撤并乡镇（街道）事业机构718个，精简事业编制680名。认真执行人大议案决议，积极办理人大代表建议和政协提案，满意率分别达99.76%和99.64%。12件22项为民办实事项目全部完成。加快行政审批制度改革，取消市级政府部门第五批行政审批事项65项。创新投资项目审批运作方式，审批时限比上年缩短1/3。落实反腐倡廉长效机制，加强行风整治，深化政务公开，加大案件查处力度，处分违纪人员586人，挽回经济损失4834万元。

过去一年我们遇到的困难比预想的还要多，我们取得的成绩比预期的还要好。一年来的实践和成就，充分展现了全市人民勇于争先、敢于胜利的可贵品质，展现了社会各界齐心合力、同舟共济的协作精神，展现了这片热土生机勃勃、蒸蒸日上的时代风采！在此，我谨代表市人民政府，向在全市各个领域、各行各业辛勤劳动、作出贡献的广大干部群众，向省部属驻泉单位和驻泉部队、武警官兵，向所有关心、支持和参与泉州现代化建设的海外侨胞、港澳台同胞、海内外朋友致以崇高的敬意！对人大代表、政协委员、民主党派、工商联、无党派人士和全市人民给予政府的高度信任和大力支持表示衷心感谢！

当前，我市经济社会运行中还存在不少困难和问题。主要表现在：一是经济发展的主要制约因素有待解决。产业结构调整不快。经济发展仍存在不少体制性、机制性障碍。多数企业技术含量偏低，自主创新能力不强。中小企业融资仍然困难。电力供应短缺，企业用地紧张。区域用工环境尚不够理想，企业经营管理人才和熟练工人缺乏。二是"三农"问题仍较突出。农业抵御自然灾害能力较弱，农民增收趋缓，城乡差距拉大，部分乡镇、村负债较重。三是经济社会发展还不相协调。人才引进、培养、使用与经济发展不相适应。高中阶段教育发展不快，对职业培训和民办教育扶持力度不够。公共卫生体系建设和农村社会事业发展滞后。四是城市化滞后于经济发展。中心城市规模偏小，功能不完善，集聚和辐射能力不强，城市现代服务业发展不快。五是环境、资源、人口压力仍然较大。一些地方环境污染和生态破坏现象还比较严重，部分地方人口出生性别结构不合理。六是政府自身建设还需加强。一些地方和部门本位主义、官僚主义、形式主义不同程度存在，干部激励约束机制需要完善，少数公务人员素质不高，服务意识差，办事效率低，投资软环境亟待进一步改善。这些问题我们要高度重视，采取切实有效措施，认真加以解决。

二、 2004 年政府工作的指导思想与总体要求

2004年政府工作的指导思想是：以邓小平理论和"三个代表"重要思想为指导，全面贯彻党的十六大和十六届三中全会精神，认真落实省委七届六次、市委九届七次全会部署，按照"五个统筹"要求，突出以人为本，坚持求真务实，创新和深化大泉州发展战略，加快建设全面、协调、可持续发展的产业竞争体系、动力促进体系、城乡互动体系、智力支持体系和生态保障体系，加快"两个率先"步伐，在建设海峡西岸经济区中作出积极贡献。根据上述指导思想，2004年我市经济社会发展的主要预期目标是：全市地区生产总值增长13%；全社会固定资产投资增长17%；社会消费品零售总额增长12%；外贸出口增长13%；实际利用外资（报表口径）增长17.3%；地方财政一般预算收入增长16%；城镇居民人均可支配收入增长8%；农民人均纯收入增长6%；城镇登记失业率控制在1.9%以内；人口自然增长率控制在6.7‰以内。实现上述目标，必须注意把握以下几点：

一要抢抓发展机遇，加快构筑泉州新一轮发展的跃升平台。强化时代紧迫感和历史使命感，精心实施、全面深化大泉州发展战略。在建设海峡西岸经济区中找准位置、凸显作用、多作贡献，在更高站位、更高层次、更高平台上实现新的起飞，保持和发展经济、文化、城市三大建设齐头并进，物质、政治、精神三大文明互动共荣，改革、发展、稳定三大关系协调统一的良好局面。

二要坚持科学发展观，努力创造全面、协调、可持续的发展格局。把握重点，统筹兼顾，促进经济、社会和人的全面发展。着力探索泉州新一轮经济发展途径，转变增长方式，提高经济素质和综合竞争力。既要抓经济增长速度，更要抓经济增长的质量与效益；既要注重经济指标，更要注重人文、资源、环境指标；既要牢固树立新发展观，更要在发展规划、政策措施、资金投入、体制改革等方面具体落实新发展观。

三要加大改革开放力度，切实增强加快发展的持久动力。充分发挥泉州作为开放地区、"民办特区"、重点侨乡、全国综合配套改革试点城市的先行探索作用，争当完善社会主义市场经济体制和加快经济发展的排头兵。加大创新力度，努力消除制约发展的体制性障碍。大胆革除与国际竞争通行规则相悖的观念意识、政策机制、管理方式，加快接轨步伐，全面提高对外开放水平。

四要坚持市县联动，着力提升整体竞争力。突出中心城市的龙头带动作用，使之切实担当起对区域资源整合、重大基础设施和产业布局、城镇体系和跨县（市、区）建设项目统一规划、统一协调等责任，提高凝聚力、带动力和辐射力。以县域经济为区域经济发展的基础，把培育特色经济和产业集群作为发展县域经济的重要途径和载体，不断壮大综合经济实力。

五要执政为民，大力营造良好的发展环境。正确处理政府与市场、公平与效率的关系，切实转变政府职能，改进工作作风，提高公共服务水平。把暖人心、得人心、稳人心的好事实事办好、办实，把服务群众、服务企业的工作做到位、做到家，真正树立起"对人民负责"和"推动工作"高度统一的崭新形象。

三、 2004 年政府工作的主要任务和措施

（一）坚持第一要务，加快建设全面、协调、可持续发展的产业竞争体系

激发产业活力。坚持工业立市。大力扩张产业集群，进一步提升石化、纺织等23个工业经济板块的发展水平，增强泉州产业集群的核心优势。加快工业结构调整和优化，抓好市100家重点企业、80家省级新经济增长点企业和65家市重点技改企业，扶持壮大1000家潜力大、产品市场前景好、产业关联度强的规模以下企业，增强工业经济发展后劲。坚持以信息化带动工业化，着力抓好"数字泉州"1118工程年度建设任务落实，深化省级电子商务示范工程建设，做好制造业信息化工程重点城市工作，引导企业走新型工业化道路。认真做好清理整合工业园区工作，以抓好13个省级工业园区为重点，培育一批精品工业园区，形成若干个关联企业集中、协作配套密切的专业化制造中心。加快港口资源整合，完善港口规划和功能布局，搞好临海工业带的规划建设，促进临海工业与港口经济协调发展。加快发展旅游服务业，以争创中国最佳旅游城市为目标，抓紧完成旅游规划修编，培育壮大旅游企业，开发特色旅游资源，全力打造"海上丝绸之路"、"惠女风情"等品牌；办好第六届泉州旅游节和2004年中国（泉州）百姓生活游系列活动。规划建设以生产资料物流、大宗商品物流为特色，以物流园区、物流基地、物流企业为依托的泉州现代物流中心，抓好奇龙物流中心、闽台农副产品对台物流中心等省布点的综合性物流配送中心建设。

增强产业后劲。全力抓好泉南沿海大通道、外走马埭围垦、洛秀组团市政配套工程、三安集团钢铁扩建工程、石狮纺织服装物流中心、南安仑苍水暖市场等65个重点项目建设，实现年度投资78.3亿元；加快启动晋江大桥、秀涂港万吨级码头、泉州船厂、晋江LNG燃气电厂、石狮PTA等39个预备重点项目；加紧泉港10万吨集装箱码头等50个重点前期项目工作。加强电源点和电网建设，加快南埔电厂建设，新建、扩建总负荷为280万千伏安的220千伏以上输变电站8座，启动县级城网改造。建设完善路网通道，争取泉三高速公路尽快建设，配合做好福厦铁路泉州段的项目前期工作。改善金融服务。加强政、银、企合作，办好第二届全市银企供需洽谈会。加快发展地方金融机构，完善农村金融服务体系，争取创办泉州农村商业银行。支持行业协会组建投资担保公司，鼓励金融机构扩大对中小企业的信贷额度和信贷品种，提高金融服务地方经济的质量。

壮大产业主体。认真实施《进一步提升民营经济发展水平的若干意见》，放宽市场准入，允许民营资本进入法律法规未禁入的基础设施、公用事业、现代服务业及其他行业和领域，鼓励支持更多民营企业申报进出口经营权。积极引导民营企业加快体制、技术、管理创新，进一步做好乡镇企业产权甄别工作，力促合伙、合作制企业改制为规范化的有限公司和股份公司，建立现代企业制度。大力支持企业加强技术创新服务体系建设，深化产品研发，做好"产、学、研"结合工作，加速科技成果转化。在抓好ISO9000、ISO14000国际标准管理体系认证的基础上，积极推进SA8000标准工作。大力实施创名牌品牌战略，加大对名牌产品的扶持力度。致力改善企业用工环境，认真落实《泉州市保障外来务工人员合法权益暂行规定》，切实维护劳动者的合法权益。实施"民营企业家素质提高工程、企业经营管理人才培养工程、熟练技术工人培训工程"，为加快我市企业上规模、上档次和转型升级服务。

优化产业布局。加快五大基地建设，努力形成块状效应。高新技术孵化基地要抓好微波通信和高科技陶瓷中试基地、市先进制造技术服务基地、中小企业ASP公共服务平台及国家级重点示范生产力促进中心建设，促进孵化基地群加快形成。民生产品制造基地要以现有20多个初具规模的板块为基础，推进连片开发，抓好25个民生产品产业基地的培育和建设。石油化学工业基地要围绕创建国家级石化园区，组织实施十大石化工程，抓紧泉港、泉惠石化工业区规划建设，做好石化中下游产业链项目的筹备和招商。特色农业产业基地要加快构建以"252"、"4121"农业基地和"1168"蓝色产业基地为主框架的产业基础骨干体系。生产要素集散基地要致力完善立体交通及水、电、通信、市政等基础设施，抓好晋江石狮纺织服装鞋业原辅材料、南安晋江建材批发、安溪茶叶等市场建设，积极培育资本、人才、劳动力、土地等各种要素市场。协调发展县域经济，围绕农业增收、工业增效、财税增长、后劲增强的目标，加快产业链和产业集群培育，进一步激发县域经济活力。推进山海产业协作，创新协作方式，强化区域产业的互动、联动效应。

（二）推进改革开放，加快建设全面、协调、可持续发展的动力促进体系

加快体制创新。深化国有资产管理监督体制改革。健全市级国有资产管理机构，完善出资人制度和监管制度。建立国有资本经营预算制度和企业经营业绩考核体系，探索国有资产监管和经营的有效形式，完善授权经营制度。继续抓好列入省级第一批战略性调整计划的7家国有工业企业的改革工作，推进市级自来水、公交、工业、建筑、旅游、外贸等行业国有企业的资产重组和结构调整。深化国有企业和城镇集体企业改革，促其成为多元持股的股份制企业。完善产权交易市场，促进各类资本的流通和重组。加快企业改制上市步伐，推进凤竹纺织、七匹狼实业顺利上市，协助浔兴拉链、冠福家用做好上市辅导验收，鼓励三安集团、泓霖化纤等一批条件成熟的企业境外上市。深化投融资体制改革，加大经营性基础设施、公用事业和社会事业领域吸纳民间资本投资力度。稳妥推进社会领域改革。加快经营性事业单位企业化、市场化、产业化。深化公益性事业单位劳动人事、收入分配和社会保障制度改革，激发其活力，提高服务水平。加快"诚信泉州"建设，组建泉州信用评估服务中心，建立信用监督和失信惩戒制度。深化行业协会试点城市工作，加快发展各类中介服务组织。加大市场经济秩序整顿力度，有重点地加强对食品、药品、房产交易和建筑装璜市场的监管，依法打击制售假冒伪劣商品和商业欺诈等违法行为。

扩大对外开放。大力开拓国际市场，确保出口持续稳步增长。认真做好广交会、华交会等国内外知名展览会参展工作。在巩固传统市场基础上，深度开发东盟市场，努力开拓俄罗斯、东欧、中东、非洲等市场。加快法兰克福泉州（德国）贸易促进中心的运作，探索在阿联酋等地设立贸易中心，支持企业"走出去"参与国际竞争。大力推进关贸、银贸、税贸、科贸合作，加强标准化体系、质量认证和检验检疫建设，重点扶持出口轻工产品检测实验室建设。完善进出口预警监测和服务机制，继续做好加入WTO后续应对工作，指导企业做好应对技术性贸易壁垒和反倾销应诉工作。落实出口退税改革政策，鼓励发展出口加工贸易。提高利用外资质量。做好招商引资项目的筛选、储备和推介工作，创立项目快速落地机制，实行供地价格与项目投资规模、技术水平、纳税额挂钩制度，提高招商引资实效。加强与跨国公司地区总部、采购中心、研发中心的交流与合作，主动吸纳台湾电子、通讯、石化、纺织、农业等产业转移，实现产业对接升级。强化重点外资建设项目的跟踪落实。重视发挥海外社团的作用，鼓励更多泉州籍乡亲回乡投资创业。把握内地与港澳建立更紧密经贸关系机遇，制定服务业对外开放的合作指南，加大对侨港澳服务业招商力度。

加强区际协作。主动对接环渤海经济圈、长江三角洲、珠江三角洲三大经济区，加大经济协作力度，提升合作交流水平。强化对内招商，发挥泉籍驻外商会的作用，引进国内大企业落户泉州。大力开拓内地城乡市场，积极主办和参加各种大型展销会、博览会，办好"北京·福建迎奥运产品展销会"。与国内友好城市开展实质性交流与合作。加快发展区域及城际信息网络，实现资源共享、联动发展。

（三）突破二元结构，加快建设全面、协调、可持续发展的城乡互动体系

致力提高城市化水平。做大做强中心城市。着眼于海峡西岸经济区中部枢纽城市和泉州湾滨海城市的发展定位，完成新一轮城市总体规划修编、市域城镇体系规划编制和洛秀组团起步区控制性详规编制，拉开大城市发展框架。抓紧做好市行政中心建筑方案与环境设计竞标工作，组织实施市行政中心前期配套工程、名城保护建设等62个城建项目，加快清濛三期、洛秀组团、江南高新技术园区建设。抓紧建设市政配套管网系统和污水、垃圾处理厂，动工建设宝洲污水处理厂二期工程，抓紧建设北峰、江南、城东、东海四个组团和南安、安溪、永春、德化四个县（市）污水处理厂，加快推进晋江、石狮等污水处理厂和垃圾焚烧厂建设。搞好城市管理经营，进一步整顿规范建筑和房地产市场，鼓励和引导民营资本进入城市基础设施建设领域。加大城市土地资源整合力度，继续实施和完善土地收储、挂牌出让制度。以创卫通过"国检"为契机，健全和落实长效管理机制。着力解决城市市容环境卫生、交通阻滞、违章建筑、拆迁安置、产权办证、物业管理、"城中村"等群众关注的热点难点问题。抓好城镇一体化规划的协调衔接，抓紧县（市）域城镇体系和小城镇总体规划修编，重点做好14个省、部级和26个市级试点镇工作。各县（市）要增强大局观念，按照大泉州发展的要求，主动对接和呼应中心城市，做到既分工明确、各展所长，又形成合力、融为一体，努力实现统一规划、整体布局、资源共享、优势互补、共同发展的"城市联盟"。各级各部门尤其是各县（市、区）要进一步消除不利于城镇化发展的体制和政策障碍，在户籍、就业、医疗、社保和公共财政等方面努力缩小城乡差别，加快"镇改街道、村改社区"工作，扎实推进城乡一体化进程。

致力解决"三农"问题。全面贯彻落实中央1号文件精神，坚持农业稳市，努力实现农业增效、农民增收、农村稳定。紧紧围绕农民增收这一主线，狠抓结构调整增收、产业化增收、科技增收、改革增收等关键环节。以发展特色、无公害农业基地为抓手，以扶持100家龙头企业创品牌为重点，推进农业产业化、标准化、外向化；抓好泉州湾星火产业带、星火技术密集区项目落实，实施"一县一业"行动示范工程；落实《关于加快农村劳动力转移的若干意见》，全年完成劳动力转移3万人以上。认真实施农村税费改革方案，强化督促检查，落实市县两级财政支出，确保改革后农民负担得到明显减轻、不反弹，确保乡镇机构和村组织正常运转，确保农村义务教育经费正常需求。继续稳定和完善土地承包经营制度，大力推进集体林权制度改革，引导土地、林地经营权流转。完善粮食预警机制，保障粮食安全。加大农业基础设施投入，完善农业防灾减灾体系，重点抓好碧坑水库、晋江下游防洪岸线整治二期工程、金鸡拦河闸改造、山美水库安全监测系统以及病险水库、海堤加固等建设。加强动植物防疫体系建设，切实抓好高致病性禽流感等禽畜病的防控工作。继续加大对安溪、永春、德化等山区的资金、项目扶持力度，实现山海协调发展。加快农村改旧建新工作，培育30个改旧建新"精品村"。按照"五通五改"要求，抓好830个行政村的环境整治。扎实推进宽裕型小康建设，新增1个县（惠安县）、34个乡镇、413个村达到宽裕型小康标准。

致力改善城乡居民生活。要关心困难群众。完善城乡居民最低生活保障制度，将全市2.83万农村贫困群众纳入低保对象，实现应保尽保；尽快解决中侨和二轻企业职工参加医疗保险、下岗失业人员再就业、退休人员社会化服务等历史遗留问题；探索就业安置、社会保险、折价入股等多样化的征地、拆迁补偿机制，维护群众的合法权益；进一步改善贫困山区、革命老区和少数民族聚居区群众的生产生活条件，抓好10个省级、15个市级扶贫开发重点村工作，支持"老、少、边、贫"自然村解决通行政村道路硬化问题；继续实施"造福工程"，搬迁安置1000人；完成安置三峡移民300人任务。致力扩大就业。落实各级政府促进就业目标责任制和再就业扶持政策，力争全年新增就业岗位13.8万个；加大职业培训力度，全年培训各类劳动力25万人以上；加大劳动关系调整力度，妥善处理劳资纠纷，依法整治拖欠工人工资问题。扩大社会保险。加强养老保险扩面工作，全年实现参保人数36.15万人；继续完善失业保险制度，全年实现参保人数38万人；完善多层次的职工医疗保险体系，全年实现参保人数30万人；以泉港、石狮为试点探索建立农村疾病医疗保险制度，以安溪为试点探索建立以"大病统筹"为主的新型农村合作医疗制度，解决农民因病返贫致贫问题。

致力维护社会安定稳定。落实安全生产责任制和重特大安全事故行政一把手责任制，深化安全生产专项整治，及时消除安全隐患，杜绝重特大事故发生。坚持"严打"方针，严厉打击严重暴力犯罪、有组织犯罪、严重经济犯罪以及盗、抢等多发性犯罪，加大对突出治安问题的重点整治力度，完善社会治安长效机制，加大防控体系建设，创建"平安泉州"。健全落实经济社会紧急情况应急处理工作预案，及时化解各种社会矛盾，努力营造和谐稳定的社会环境和公正高效的法治环境。

（四）弘扬先进文化，加快建设全面、协调、可持续发展的智力支持体系

推动人才强市迈出新步伐。坚持人才强市，加大人才工作力度，抓紧出台我市加强人才工作的政策措施。抓住培育、吸引、用好人才三个环节，壮大经济社会发展亟需的科技专业人才、企业家、公务员和技工人才队伍。加强博士后工作站、高校创业园、留学生创业园等建设，大力营造有利于人才成长和创业的环境，形成多层次、全方位、系统化的人才开发新格局。

推动科教兴市开创新局面。加快科技进步。完善以泉州高新技术孵化基地、企业博士后科研工作站及科研院所为依托的科技创新体系，鼓励开发具有自主知识产权和市场竞争力的新技术、新产品。推进四大国家级科技示范工程建设，组织实施各级各类科技计划100项。加快教育发展。建立以"一增三保"为目标的农村义务教育经费投入保障机制，加快农村中小学危房改造。全面启动"双高普九"。大力推进高中阶段和职业技术教育，提高高中普及率和素质教育水平，继续推进职业教育。积极为华侨大学、仰恩大学、黎明大学等院校创造良好的办学条件。抓紧泉州高教发展中心和泉州师院东海校区建设，力争医高专获批、泉州师院通过教育部教学工作水平评估，为筹建泉州大学奠定基础。全面落实《民办教育促进法》，大力鼓励和扶持民办教育发展。支持申办福建光电学院、泉州化工职业技术学院、泉州纺织职业学院、泉州幼儿师范专科学校、永春职业技术学院。

推动社会事业实现新突破。繁荣文化事业。做好"海丝"申报"世遗"迎检工作和南音申报"人类口头及非物质遗产代表作"工作，继续办好"中国南音年"活动。传承保护好梨园戏、木偶、南音等传统艺术，推动开展"泉州学"研究。加强艺术精品创作和生产，力争国家舞台精品工程初选剧目《董生与李氏》入选全国"十大精品剧目"。抓紧梨园古典剧院、南建筑博物馆等一批市级重点社会事业项目建设。深入开展"创建文化先进县"活动，巩固发展四级文化网络。继续深化文化体制改革和文化表演团体改革，扩大对外、对港澳台文化交流与合作。发展卫生事业。加强公共卫生体系建设，及时控制和消除各类突发性公共卫生事件。建设市传染病医院，完成市、县两级卫生监督体制和疾病控制体制改革。加强农村卫生工作，增加农村卫生投入。落实全民健身计划，大力开展群众性体育活动，提高竞技体育水平，壮大体育产业，启动承办第六届全国农民运动会各项准备工作。认真实施《泉州市妇女发展纲要（2001-2010年）》和《泉州市儿童发展纲要（2001-2010年）》，加快发展广播影视、新闻出版、社会科学、老龄、残疾人、关心下一代、档案、民族、宗教、气象、地方志、抗震减灾等社会事业。

推动精神文明建设取得新成效。坚持不懈地推进"八城"创建活动，全面提高公民文明素质和城乡文明程度。认真贯彻《公民道德建设实施纲要》，深入开展群众性精神文明创建活动。普及科学知识，反对封建迷信，倡导健康文明的生活方式。着重培育一批各具特色的新型社区典型，带动社区建设全面发展。加大扫黄打非力度，保护知识产权，整顿文化市场。

推动国防动员和双拥工作再上新台阶。加强国防教育，抓好民兵、预备役部队建设，扎实做好国防动员工作。妥善安置退伍、转业军人和随军家属。支持部队国防工程建设，加强军事设施保护。以荣获全国双拥模范城"五连冠"为新的起点，推动双拥工作进企业、进机关、进学校、进社区、进连队，为加强国防建设作出新的贡献。

（五）贯彻三大国策，加快建设全面、协调、可持续发展的生态保障体系

提高计划生育工作水平。以稳定低生育水平、提高人口出生素质、促进人的全面发展为目标，深化人口与计划生育综合改革，建立健全"依法管理、村（居）民自治、优质服务、政策推动、综合治理"的人口与计划生育工作新机制。落实各级人口与计划生育目标管理责任制，开展创建优质服务县（市、区）活动。发挥计生协会"带头、宣传、服务、监督、交流"功能。全年人口出生率控制在12‰以下，政策符合率达到91.5%以上。

提高资源保护开发水平。鼓励全社会节约资源，推进清洁生产，发展循环经济。实行最严格的耕地保护制度，保持耕地占补平衡，加强土地管理，做好土地利用总体规划修编工作，合理调整城乡建设用地结构。实施海洋功能区划，依法管理海洋。加强对泉州滨海资源和泉州湾海域水质和生态的保护。合理利用和配置水资源，改善南、北渠饮用水源水质。进一步加强矿产资源管理，做好地质灾害防治工作。完善台风、洪涝、干旱、地震等特大灾害的预报、预警机制，加强防灾、减灾、救灾工作。

提高生态环境建设水平。制定实施"创建国家环保模范城市"2004年工作计划。认真执行市十三届人大二次会议2号议案决议，落实两江流域工业污染源综合治理"零点计划"，巩固整治成果。抓好《2003年-2005年泉州市污染防治工作实施方案》落实，促进环境长效管理。继续开展区域性和行业性环境综合整治工作。加强各级自然保护区和生态示范区建设。深入贯彻一系列市政府令，封山育林，保持水土，优化生态，美化环境。深入开展治山治水治田治村治矿活动，全面保护"四山两江"，努力建设一个良性循环和可持续发展的大泉州生态环境。

四、建设公共服务型政府

执政为民，字字千钧。实现人民的意愿，让广大群众在发展中得到更多的实惠，政府责无旁贷!各级政府要坚持环境活市，致力于提高公共服务能力，为经济社会发展提供足够的公共资源和良好的外部环境，为全面建设小康社会提供优质高效的服务，向人民交上一份满意的答卷。

坚持公开行政，强化公共服务。完善政务公开制度，拓宽群众获取政务信息、向政府建言献策的渠道，保障人民群众的知情权和参与权，自觉接受人民的监督，建设"阳光政府"。加快电子政务建设，做好"中国泉州"政府网站信息公布工作，抓好市直政务横向接入网建设，试行网上并联审批，拓宽为民服务领域。完善公共财政框架，规范公共支出范围，增强提供公共卫生、义务教育、社会保障、环境保护等公共产品和服务的能力。

坚持为民行政，维护群众利益。从群众最关心、最迫切需要解决的实际问题入手，诚心诚意办实事，尽心竭力解难事，坚持不懈做好事。认真落实好为民兴办的11件26项实事项目，取信于民。加强诚信政府建设，说到做到，抓好落实。深入开展机关效能建设，加大明察暗访、社会评议和告诫惩处监督力度，提高政府为民办事效率。

坚持科学行政，强化责任落实。完善政府重大经济社会问题决策程序，逐步规范咨询、论证、审议、公布、备案和听证等各个环节，加强决策监督，推进政府决策科学化、民主化、法制化。夯实决策基础。完善统计管理体制，健全经济运行监测体系，做好第一次全国经济普查工作。启动"十一五"计划的研究制订工作。加大培训教育力度，提高公务员素质，建设学习型政府。建立政府系统目标责任制，明确绩效考评办法，把决策落到实处。

坚持依法行政，规范行政行为。深化"四五"普法，全面启动依法治市中期目标的各项工作，推进"依法治市"进程。认真实施《行政许可法》，按时完成各项清理工作，规范行政许可行为。加强综合执法，全面推行办案责任制、公示制、评议考核制、过错责任追究制，做到有权必有责、用权受监督、侵权要赔偿。深化人事制度改革，推进公务员考试录用、竞争上岗制度改革，继续做好市县乡机关人员分流工作。

坚持廉洁行政，树立良好形象。认真落实《中国共产党党内监督条例（试行）》、《中国共产党纪律处分条例》和反腐倡廉"六个机制"，从源头上、制度上预防和治理腐败。把维护群众利益作为反腐倡廉的工作重点，着力解决损害群众利益的突出问题。继续抓好大案、要案的查处，严厉打击和清除腐败分子。坚决纠正部门和行业不正之风。牢记"两个务必"，筑牢思想防线，提高拒腐防变和抵御风险的能力。

时代赋予我们重任，人民寄予我们厚望。我们要倍加珍惜历史机遇，倍加珍惜泉州心齐、气顺、劲足的大好局面，在市委领导下，自觉接受市人大及其常委会监督，完善向人大及其常委会报告工作的制度，认真执行人大及其常委会的决议、决定，积极支持政协履行政治协商、民主监督和参政议政职能，完善政府与政协工作联系制度，认真听取各民主党派、工商联、无党派人士以及专家学者的意见，及时办理人大代表建议、批评、意见和政协提案，发挥工会、共青团、妇联、侨联、科协、文联、社科联、台联、金联、残联、关工委、计生协会、老龄委等群团组织作用，凝聚全市上下海内海外的智慧和力量，共同推进我市改革和发展大业.

各位代表！完善社会主义市场经济体制，全面建设小康社会，这是前无古人的伟大实践和历史跨越。重任在肩，时不我待！让我们在省委、省政府和市委的坚强领导下，全面贯彻落实党的十六大和十六届三中全会精神，以饱满的热情、昂扬的斗志、创新的精神、扎实的工作，同心同德，艰苦奋斗，共同创造泉州更加美好的明天！
